# Longtime Neurologic Outcome of Extracorporeal Membrane Oxygenation and Non Extracorporeal Membrane Oxygenation Acute Respiratory Distress Syndrome Survivors

**DOI:** 10.3390/jcm8071020

**Published:** 2019-07-12

**Authors:** Lars-Olav Harnisch, Sebastian Riech, Marion Mueller, Vanessa Gramueller, Michael Quintel, Onnen Moerer

**Affiliations:** 1 Department of Anesthesiology, University Medical Center Goettingen, 37075 Goettingen, Germany; 2 Interdisciplinary Department of Emergency Medicine, University Medical Center Goettingen, 37075 Goettingen, Germany; 3 St. Josefs-Hospital Cloppenburg, Department of Anesthesia & Intensive Care, Krankenhausstr. 13, 49661 Cloppenburg, Germany; 4 Hospital Stuttgart, Department of Neurology, Kriegsbergstr. 60, 70174 Stuttgart, Germany

**Keywords:** extracorporeal circulation, ECMO, acute respiratory distress syndrome (ARDS)

## Abstract

Neurologic complications following acute respiratory distress syndrome (ARDS) are well described, however, information on the neurologic outcome regarding peripheral nervous system complications in critically ill ARDS patients, especially those who received extracorporeal membrane oxygenation (ECMO) are lacking. In this prospective observational study 28 ARDS patients who survived after ECMO or conventional nonECMO treatment were examined for neurological findings. Nine patients had findings related to cranial nerve innervation, which differed between ECMO and nonECMO patients (*p* = 0.031). ECMO patients had severely increased patella tendon reflex (PTR) reflex levels (*p* = 0.027 vs. *p* = 0.125) as well as gastrocnemius tendon reflex (GTR) (*p* = 0.041 right, *p* = 0.149 left) were affected on the right, but not on the left side presumably associated with ECMO cannulation. Paresis (14.3% of patients) was only found in the ECMO group (*p* = 0.067). Paresthesia was frequent (nonECMO 53.8%, ECMO 62.5%; *p* = 0.064), in nonECMO most frequently due to initial trauma and polyneuropathy, in the ECMO group mainly due to impairments of N. cutaneus femoris lateralis (4 vs. 0; *p* = 0.031). Besides well-known central neurologic complications, more subtle complications were detected by thorough clinical examination. These findings are sufficient to hamper activities of daily living and impair quality of life and psychological health and are presumably directly related to ECMO therapy.

## 1. Introduction

Acute Respiratory Distress Syndrome (ARDS) is a life-threatening condition [1]. Standard therapy includes the application of ventilatory settings that have been proven to limit the secondary injury caused by mechanical ventilation, adequate positive endexpiratory pressure (PEEP), prone positioning, and the treatment of remediable causes [2]. These measures are intended to gain the patients lung time to recover while at the same time not adding further harm, which is termed ventilator-induced lung injury (VILI) [3]. Extracorporeal membrane oxygenation may be employed in severe hypoxemic respiratory failure unresponsive to standard care [4,5].

The use of ECMO has dramatically increased worldwide in recent years [6]; due both to enhanced experience and developed technology leading to widened indications and an increasing number of hospitals utilizing this technique. Though the technique has become almost standard care as a rescue therapy in patients with respiratory failure when less invasive measures have failed, complications related to the technique should be considered and weighted against its benefits [5]. Common neurologic complications attributed to the use of ECMO and the need for anticoagulation are intracranial hemorrhage and stroke [7,8,9]. Although their incidence has decreased over time [7,10], they remain common [9,10]. Moreover, the full range of complications might be underreported because emphasis is understandably put on the more apparent and debilitating sequelae. Nevertheless, subtle neurologic impairments can negatively influence the quality of life and prevent successful rehabilitation [11].

The goal of our study was to examine ARDS survivors for pathologies in the clinical examination (primary outcome) and to compare the subgroups of ECMO vs. nonECMO management concerning the incidence of neurologic findings (secondary outcome). We hypothesized that neurologic findings were a frequent finding but were not associated with the use of ECMO.

## 2. Materials and Methods

We conducted a prospective cohort study of severe ARDS-patients who survived until discharge from the intensive care unit (ICU). Patients were recruited from the anesthesiologic ICUs of Goettingen University Medical Center, a tertiary medical center, between the years 2014 and 2016. This study was approved by the University’s Research Ethics board (IRB No. 10/12/12) and written informed consent was obtained from all subjects prior to examination.

All identified patients were invited to visit our department for a follow-up assessment. 

The follow-up visit consisted of:

- a thorough medical history of the time from hospital discharge up to the day of the visit

Neurological examination consisted of:

- assessment of orientation
- clinical assessment of cranial nerve function
- muscle strength
- sensation
- motor activities
- coordination (finger-to-nose-test, rapid alternating movements), testing for postural instability (Rhomberg test, pronator-drift), and walking the patient in different modalities (normal, tiptoe, tightrope).

For the walking test, we pooled all modes and rated the test pathologic if at least one of the different modes was pathologic.

Neurologic constraints that were preexisting were recorded but excluded from analysis to be able to attribute the findings to the time of hospitalization in which ARDS occurred.

Statistical analysis was performed using SPSS (International Business Machines Corporated [IBM], Armonk, NY, USA versions 24.0 and 25.0), tests exercised were Pearsons Chi2, Fishers exact test and Welch test where eligible; statistical significance was assumed at the five percent level.

## 3. Results

We identified 144 patients who survived until ICU discharge. Of these 144 patients, 37 (26%) had died since discharge, another 69 patients (48%) were not accessible for unknown reasons. We were able to follow-up with 38 patients (ECMO: 21, nonECMO 17); of these, none refused attendance but ten were unable to visit our center (physical disability: five, psychological disability: two, moved far away: three) leaving 28 patients for examination and analysis. All of these patients were cannulated via the femoral and internal jugular veins. Patient characteristics are displayed in Table 1. All patients received passive physiotherapy from day one and active physiotherapy as soon as possible. No complications other than coagulation abnormalities, which is a known complication of ECMO-therapy were experienced.

The findings of neurological testing are displayed in Table 2.

All assessed patients were oriented to person, place and time except for one patient who was disoriented in terms of time. This patient was 82 years of age and from the results of neuropsychological testing reported elsewhere we strongly suspected that he was in the initial stage of dementia although he was not formally diagnosed at the time of follow-up.

Supplementary Table S1 shows in detail the findings of the neurologic examination (See ESM file). In nine patients there were findings related to cranial nerve integrity (4 nonECMO, 5 ECMO, *p* = 0.604). All of the findings in patients managed without ECMO were due to initial trauma leading to hospital admission. Among patients managed with ECMO we found one patient with nystagmus while following an object with their eyes, one patient had diplopia, two patients were found to have central nervus facialis palsy, and one patient complained of hyposmia and reduced taste. Three patients had preexisting conditions which were recorded but excluded from statistical analysis. After correction (exclusion of preexisting constraints), differences were statistically significant (*p* = 0.031). 

Findings of the Rhomberg test as well as pronator-drift were not different between groups.

Walking was found to be abnormal resulting in a positive test result in 13 patients representing 45% of all patients; again, no differences between groups were detected (*p* = 1.0).

The test of rapid alternating movements was positive in only one patient in the nonECMO group who suffered a stroke after recovery from ARDS. The finger-to-nose-test was untestable in two patients, who had ankylosed joints (shoulder, elbow respectively). We scored this test negative because the test was negative with the contralateral side, as were all other tests of coordinative function (rapid alternating movements, walking) in both cases.

**Figure 1:**
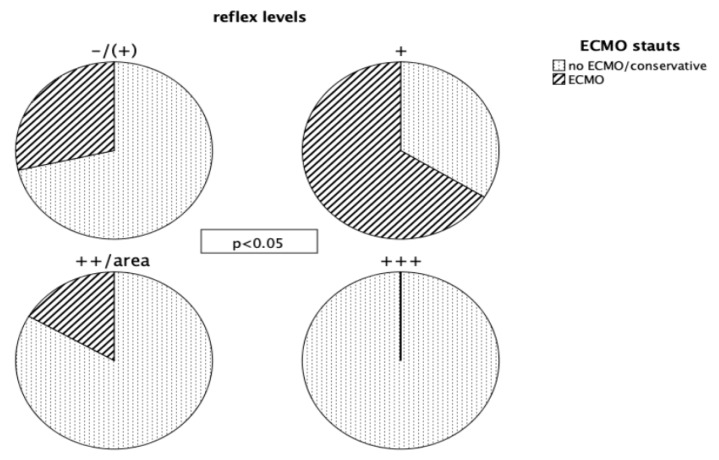
Displayed are the different reflex levels we found. “+” denoting a normal reflex level, “-/ (+)” denoting absent/reduced reflexes, “++/area” denoting an increased reflex level, an extended area to trigger a reflex respectively, “+++” denoting a very increased reflex level. The bulk of patients managed with ECMO had normal reflex levels, whereas reduced as well as increased reflex levels were mainly found in conservatively managed patients. Very increased reflex levels were invariably found in patients managed without ECMO; differences were statistically significant.

Tendon reflex differences were large but only increased reflex levels were statistically significant (biceps tendon reflex: nonECMO vs. ECMO, Fisher *p* < 0.05; triceps tendon reflex: nonECMO vs. ECMO, *p* < 0.05; patellar-tendon reflex: nonECMO vs. ECMO *p* = 0.027) (Figure 1); no pathologic reflexes (Babinski) were found.

Paresis were found in 17.8% of patients, after correction for preexisting constraints (one herniated vertebral disc) paresis were found exclusively in the ECMO group (*p* = 0.044).

**Figure 2:**
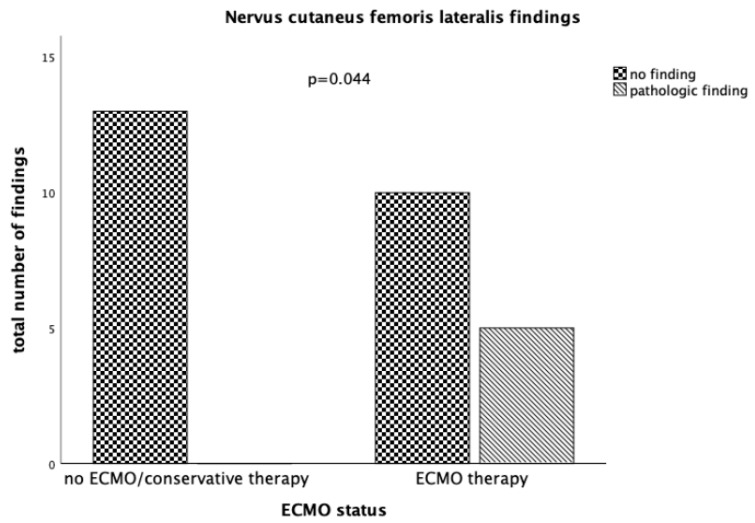
Displayed are the examination results regarding findings in the nervus cutaneus femoris lateralis separated by ECMO status. In the conservative therapy group there were no pathologic findings regarding this nerve, whereas in the group of patients managed with ECMO a third of patients had pathologic findings in the supply area of this nerve. The differences between those two groups are statistically significant.

Paresthesia was frequent in both groups (total 58.6%, nonECMO 53.8%, ECMO 62.5%; *p* = 0.128). Paresthesia in the group managed without ECMO was most frequently due to initial trauma (three cases) or polyneuropathy (critical illness, anticonvulsive medication, diabetic, one case each), whereas in the group managed with ECMO, paresthesia was often found due to impairments of nervus cutaneus femoris lateralis function (5 vs. 0; *p* = 0.044) (Figure 2).

## 4. Discussion

Neurologic impairments are frequent during ECMO therapy [7,8,9,12,13,14,15], mainly affecting the central nervous system such as ischemic stroke (IS) or intracerebral hemorrhage (ICH). These complications lead to increased ICU and hospital stay, morbidity, and mortality [16,17,18]. Critical-illness polyneuropathy and ICU-AWS (intensive care unit acquired weakness syndrome) both affecting mainly the peripheral nervous system are almost ubiquitous following critical illness [19,20]. Patients usually recover from these syndromes to some degree; however, incomplete recovery and persistent impairments are common [21]. Although impairments in these areas are by far not as devastating as ICH or IS from a medical point of view, decreased sensitivity, spasticity, or limitations in motor skills probably reduce quality of life [22,23].

Of the cranial nerve lesions in the nonECMO group, all can be explained by the underlying trauma. Lesions in the ECMO group can nevertheless be explained. The nystagmus was consistent with an ophthalmoplegia internuclearis anterior, a lesion of the fasciculus longitudinalis medialis. Causes for this lesion are mainly trauma or ischemia, both were not applicable in our case. Although an idiopathic ophthalmoplegia internuclearis anterior has been described [24], the most likely explanation is a small ischemic/hypoxic lesion. Hyposmia can be a consequence of infection; this is admittedly an infrequent but not unusual finding unrelated to ECMO use [25]. Facial nerve palsy can be caused by febrile illness in rare cases [26]. Diplopia can again be caused by ophthalmoplegia internuclearis anterior, which would be the second patient in our cohort.

Within our cohort nearly half of the patients had trouble walking in a stable manner when challenged. To our knowledge this finding has not been described before in the literature. In their prospective observation, Mehrholz et al. reported that many of their patients showed impaired walking compared to the age adjusted control [11]. In our cohort, with a mean follow-up time of 23 months, findings were most likely residues of ICU-AWS. This explanation was supported by the negative coordinative tests, suggesting that the underlying pathology was probably not central but rather a problem of peripheral nerves and postural reflexes as can be found in ICU-AWS [19].

We found that most patients had normal tendon reflexes and increased reflexes were found exclusively in the conservatively managed group. Hyperreflexia is usually conjoined with spasticity in corresponding muscles and is caused by a lesion of the upper motor neuron; the lower neuron needs to be intact, otherwise flaccid paralysis would result. Pathophysiologically, spasticity and increased reflexes are explained as the disinhibition of spinal reflexes due to lost inhibition of the upper motor neuron. Usually, pathologic reflexes of the Babinski group go along with an increased reflex level. In our study we found significantly increased reflexes in the conservative group, nevertheless, we did not find any pathologic reflexes or spasticity. 

In animal models hyperreflexia has been described as a marker of neuronal recovery [27,28]. Although not formally diagnosed but due to the severity of illness, we can rightfully assume that all of the examined patients had critical illness polyneuropathy with some degree of nerval damage. During the phase of rehabilitation, spinal recovery might have led to dendritic dysgenesis resulting in hyperreflexia without spasticity or pathologic reflexes. This explanation, although hypothetical, would perfectly explain our findings. Interestingly, this condition could be prevented by specialized physiotherapy or even pharmacologically [29,30].

Of the four patients who were found to have paresis, three had generalized weakness of the upper extremities and one patient had reduced strength in foot flexors and extensors on the right side. We do not attribute this finding to ECMO cannula because damage of the sacral plexus or sciatic nerve are almost impossible using a percutaneous ultrasound-guided cannulation technique. Furthermore, cannulation was not found to be difficult in this case.

Paresthesia was a frequent finding in our patients. It has not extensively been described in critically ill patients in general, but only in conjunction with critical illness polyneuropathy. Paresthesia in the group of conservatively managed patients could be explained by initial trauma or polyneuropathy. In the group of patients managed with ECMO, paresthesia of the right nervus cutaneus femoris was the most common finding. Since all patients were cannulated via the right femoral vein, it stands to reason that the lesions were related to compression of the nerve by the ECMO cannula. It should be noted that we found these lesions, although times on ECMO differed markedly between patients. No difficult cannulation was reported for any patient.

Due to anatomic variations the position of the nerve is not always distant from the insertion site of the cannula and we do not routinely identify the nerve by ultrasonography. The probable mechanism of this type of nerve injury is axonotmesis caused by compression of the nerve as described by Suddon [31]. In this type of injury, the actual nerve fibers are interrupted whereas surrounding tissue (endoneurium, perineurium) is preserved and may facilitate complete nerve recovery. For our cohort this would mean that a lot more patients may have had pressure injury, but many of them recovered and were not detectable at the time of follow-up. The cases where we still found the lesions might have had neurotmesis according to Suddon [31]. In these cases, the pressure on the nerval structure led to disruption of vascular supply long enough to cause necrosis of the accompanying structures of the nerve fibers and therefore a physiologic disruption (conduction failure) of all axonal and endoneurial elements of the nerves [32,33]. No clear-cut time after which permanent nerve damage happens can be found in scientific literature; even short periods of about 60 min of pressure have been described to cause nerve damage [34]. Since, recovery did not happen at follow-up, nearly 24 months after removal of the cannula, recovery is very unlikely [35].

The main limitation of our study was the severe loss of patients for follow-up. One possible explanation would be that we tried to contact patients after a variable period after discharge without them knowing we would. Moreover, not all contacted patients were able to attend the follow-up visit mainly due to psychological reasons (e.g., claustrophobia, panic attacks, severe anxiety), patients needing extensive care were also excluded from the study. Therefore, the true number of neurological sequelae might be even higher than reported here.

## 5. Conclusions

Survivors of ARDS show neurologic complications related to the disease or the necessary (rescue-) therapy. Besides well-known complications like intracranial hemorrhage and ischemic stroke, even more subtle lesions can be detected by a thorough clinical examination. These findings are arguably not as severe as the aforementioned, nevertheless sufficient to hamper activities of daily living and work and therefore impair quality of life and psychological health. Long-term neurologic complications directly related to ECMO therapy should not be ignored, especially when considering ECMO for indications with unproven evidence, such as less severe ARDS or severe ARDS without having established all conservative measures such as prone positioning and adequate PEEP [5]. Due to the “pilot-trial character” of this study assessing a retrospective set of patients for a prospective observation will require a large number of unknown cases.

## Figures and Tables

**Table 1:** Patient characteristics, values are displayed as mean ± standard deviation, significant differences are marked *.

	ECMO	nonECMO	*p*-Value
Age	53.87 ± 10.66	61.92 ± 10.81	0.059
Sex (f:m)	6:11	5:8	0.526
SAPS II admission	33.80 ± 9.34	37.69 ± 10.74	0.320
ECMO duration (days)	11.26 ± 8.22	-	-
Mechanical ventilation (days)	17.86 ± 12.69	9.95 ± 5.18	0.046 *
ICU stay (days)	20.93 ± 11.34	19.69 ± 11.18	0.773
Hospital stay (days)	40.93 ± 16.57	39.38 ± 24.61	0.849
Discharge – Follow-up (months)	21.85 ± 12.13	23.82 ± 11.47	0.628

**Table 2:** Summarized findings of neurologic examination by treatment group. Any abnormal finding was counted, findings of patients were added.

	ECMO	nonECMO	*p*-Value
Orientation (no. of findings)	0	1	0.464
Cranial nerves (no. of findings)	5	4	1.0
Rhomberg test (no. of findings)	4	2	0.655
Arm elevation (no. of findings)	3	0	0.226
Walking (no. of findings)	7	6	1.0
Diadochokinesis (no. of findings)	0	0	-
Finger-nose-test (no. of findings)	0	0	-
BTR right (no. of findings)	1	5	0.013 *
BTR left (no. of findings)	0	5	0.013 *
TTR right (no. of findings)	1	5	0.022 *
TTR left (no. of findings)	0	5	0.013 *
BRTR right (no. of findings)	0	4	0.035 *
BRTR left (no. of findings)	0	4	0.035 *
PTR right (no. of findings)	3	9	0.027 *
PTR left (no. of findings)	4	8	0.125
GTR right (no. of findings)	2	8	0.041 *
GTR left (no. of findings)	2	7	0.149
Babinski (no. of findings)	0	0	-
Paresis (no. of findings)	5	0	0.044 *
Paresthesia (no. of findings)	10	4	0.128
Lesion of N. cutaneus femoris lateralis (no. of findings)	5	0	0.044 *

Significant differences are marked *. Abbreviations: BTR – biceps tendon reflex; TTR – triceps tendon reflex; BRTR – brachioradialis tendon reflex; PTR – patella tendon reflex; GTR – gastrocnemius tendon reflex.

## Supplementary Materials

The following are available online at https://www.mdpi.com/2077-0383/8/7/1020/s1.
